# *Ilex paraguariensis* A.St.-Hil. improves lipid metabolism in high-fat diet-fed obese rats and suppresses intracellular lipid accumulation in 3T3-L1 adipocytes via the AMPK-dependent and insulin signaling pathways

**DOI:** 10.29219/fnr.v68.10307

**Published:** 2024-01-22

**Authors:** Maya Kudo, Ming Gao, Misa Hayashi, Yukiko Kobayashi, Jinwei Yang, Tonghua Liu

**Affiliations:** 1 School of Pharmacy and Pharmaceutical Science, Mukogawa Women’s University, Nishinomiya, Hyogo, Japan; 2 Institute for Bioscience, Mukogawa Women’s University, Nishinomiya, Hyogo, Japan; 3 Tokiwa Phytochemical Co., Ltd., Sakura, Chiba, Japan; 4 Key Laboratory of Health Cultivation of the Ministry of Education, Beijing University of Chinese Medicine, Beijing, China

**Keywords:** mate, life-style-related diseases, adipose tissue, adipogenesis, lipolysis, lipid metabolism pathway

## Abstract

**Background:**

Obesity is closely associated with several chronic diseases, and adipose tissue plays a major role in modulating energy metabolism.

**Objective:**

This study aimed to determine whether Mate, derived from *I. paraguariensis* A.St.-Hil., ameliorates lipid metabolism in 3T3-L1 adipocytes and high-fat diet (HFD)-fed obese Sprague-Dawley (SD) rats.

**Design:**

3T3-L1 adipocytes were cultured for 7 days, following which intracellular lipid accumulation and expression levels of lipid metabolism-related factors were examined. Dorsomorphin was used to investigate the potential pathways involved, particularly the adenosine monophosphate-activated protein kinase (AMPK)- dependent pathway. Mate was administered to rat HFD-fed obese SD models for 8 consecutive weeks. The expression of lipid metabolism-related factors in the organs and tissues collected from dissected SD rats was evaluated.

**Results:**

Mate suppressed intracellular lipid accumulation in 3T3-L1 adipocytes, increased the protein and gene expression levels of AMPK, hormone sensitive lipase (HSL), calmodulin kinase kinase (CaMKK), liver kinase B1 (LKB1), protein kinase A (PKA), CCAAT/enhancer binding protein *β* (C/EBP*β*), insulin receptor b (IR*β*), and insulin receptor substrate 1 (IRS1) (Tyr465), and decreased those of sterol regulatory element binding protein 1C (*Srebp1c*), fatty acid synthase (FAS), peroxisome-activated receptor γ (PPARγ), and IRS1 (Ser1101). Furthermore, an AMPK inhibitor abolished the effects exerted by Mate on intracellular lipid accumulation and HSL and FAS expression levels. Mate treatment suppressed body weight gain and improved serum cholesterol levels in HFD-fed obese SD rats. Treatment with Mate increased the protein and gene expression levels of AMPK, PKA, Erk1/Erk2 (p44/p42), and uncoupling protein 1 and reduced those of mammalian target of rapamycin, S6 kinase, *Srebp1c, ap2*, FAS, *Il6, Adiponectin, Leptin*, and *Fabp4* in rat HFD-fed obese SD models.

**Discussion and conclusions:**

Mate suppressed intracellular lipid accumulation in 3T3-L1 adipocytes and improved lipid metabolism in the epididymal adipose tissue of HFD-fed obese SD rats via the activation of AMPK-dependent and insulin signaling pathways.

## Popular scientific summary

- Mate suppressed intracellular lipid accumulation and improved lipid metabolism
- Mate inhibited body weight gain, reduced serum cholesterol levels by inhibiting adipogenesis, and promoted lipolysis via the AMPK-dependent pathway
- Mate shows potential for suppressing the onset of various lifestyle-related diseases

Obesity, long considered a public health concern worldwide, is a major risk factor for diseases such as type 2 diabetes (T2D), cardiovascular disease, and non-alcoholic fatty liver disease (NAFLD) (1–3). However, due to the adverse side effects exerted by currently recommended medical treatments, the options left for treating patients with obesity remain limited (4, 5). Studies have shown that many natural resources containing active phytochemicals that exert beneficial biological effects are useful as therapeutic agents for obesity (6).

White adipose tissue (WAT) stores large amounts of triglycerides (TG) as chemical energy in unilocular droplets, which are released into circulation as needed (7) and produce hormones and cytokines that regulate the immune system (8). In this study, we focused on epididymal adipose tissue (EAT), a type of WAT. An increase in WAT mass is directly associated with an increased incidence of metabolic disorders, such as obesity and T2D (9). Lipid accumulation in WAT is regulated by lipogenesis and lipolysis pathways, which involve various transcription factors, genes, enzymes, and proteins (10).

Adenosine monophosphate-activated protein kinase (AMPK) is an important regulator of energy metabolism in multiple tissues and adipocytes, and the AMPK-dependent pathway is a crucial pathway associated with lipid metabolism (11, 12). AMPK reportedly inhibits the synthesis of fatty acids, cholesterol (TC), and isoprenoids as well as hepatic gluconeogenesis while increasing muscle glucose transport, fatty acid oxidation, and calorie intake (11). AMPK is activated via the phosphorylation of upstream kinases, including liver kinase B (LKB) and calmodulin kinase kinase (CaMKK) (13, 14, 15). Approximately 40 years ago, it was discovered that AMPK was regulated by acetyl-CoA carboxylase (ACC) activity (16). ACC plays an important role in fatty acid synthesis and oxidation (17). Fatty acid synthase (FAS), a downstream factor of AMPK, is targeted by the transcription factor sterol regulatory element binding protein 1C (SREBP1C) (18). SREBP1C is an important regulator of adipogenesis (19). Adipogenesis is defined as the process by which preadipocytes differentiate into adipocytes (4). Differentiation of preadipocytes into adipocytes is regulated by the expression of proteins that induce mature adipocyte formation and the complex interactions between transcription factors such as CCAAT/enhancer binding protein α (C/EBPα) and peroxisome-activated receptor γ (PPARγ) (19). C/EBP activates PPARγ expression during the early stages of differentiation (20), and PPARγ promotes the expression of genes related to lipogenesis, while SREBP1C induces ACC and FAS (21). In addition, adipocytes of individuals with obesity secrete inflammatory cytokines, such as tumor necrosis factor α (TNFα) and interleukin-6 (IL-6), which induce an inflammatory state. AMPK is also involved in the regulation of these cytokines and hormones, making it a promising molecular target for drugs aimed at treating metabolic disorders, including obesity (4).

The insulin signaling pathway regulated insulin-stimulated intracellular lipid production in 3T3-L1 adipocytes (22, 23). Insulin receptor (IR) pathway-related proteins, such as IR, Erk1/Erk2 (p44/p42) (Erk), protein kinase B (Akt), and jun amino terminal kinase (JNK), are affected by insulin (24–26). The insulin signaling pathway also contributes to the expression of adipogenic markers such as C/EBPα and PPARγ, via the phosphorylation of Akt (25). Therefore, the insulin signaling pathway may also provide a target in treating obesity.

*Ilex paraguariensis* A.St.-Hil. (Mate) is an evergreen tree of the Aquifoliaceae family that grows naturally in subtropical forests near the Atlantic Ocean (Brazil, Paraguay, and Argentina); it is native to the area around the Iguazu waterfall (26–28). Mate, derived from *I. paraguariensis* A.St.-Hil., is rich in caffeine, iron, and calcium, has a high nutritional value, and facilitates recovery from physical and mental fatigue. Mate contains polyphenols (chlorogenic acid, caffeic acid, 3,4-dicaffeoylquinic acid, and 3,5-dicaffeoylquinic acid), xanthines (caffeine and theobromine), flavonoids (quercetin, kaempferol, and rutin), amino acids, minerals (phosphorous, iron, and calcium), and vitamins (C, B1, and B2) (29–32). The effects exerted by Mate on animal models have been extensively reported (33–38). According to recently published research, Mate extract prevents hypolipidemia (34, 39), protects lipoprotein antioxidants (40–45), and suppresses atherosclerosis (46–48). Although Mate extract has been linked to anti-obesity effects (26, 49, 50), evidence supporting this hypothesis remains scant (39, 51).

We have previously studied the effects of natural resources on lifestyle diseases such as obesity and diabetes (51–58).

L-citrulline exerts anti-obesity effects that suppress appetite in rat high-fat diet (HFD)-fed models (52) and inhibits hepatic fat accumulation by improving lipid metabolism in rat NAFLD models (56). *Morinda citrifolia* (Noni) fruit juice prevented stroke by promoting the production of nitric oxide in SHRSP rats (53), while madecassoside, plasmalogen, and amycenone reduce body weight by promoting lipid metabolism pathway in KK-*A^y^* mice (55, 57, 58). Furthermore, YNCRG-based health foods developed and blended in our laboratory inhibited the metabolic syndrome via appetite suppression and improved lipid metabolism in rat metabolic syndrome models (54).

In this study, we investigated the suppression of intracellular lipid accumulation in 3T3-L1 adipocytes and the enhancement of lipid metabolism in HFD-fed obese Sprague-Dawley (SD) rats induced by Mate via the AMPK-dependent and insulin signaling pathways.

## Material and methods

### Ilex paraguariensis *A.St.-Hil. (Mate) extract preparation*

Powdered Mate extract (MATESOL^TM^) was manufactured by Tokiwa Phytochemical Co., Ltd (Chiba, Japan). Dried leaves of *I. paraguariensis* A.St.-Hil. (10 kg) were extracted twice with 100 L of water. The extract was added, adsorbed, filtered, evaporated, and spray-dried to obtain a powdered extract (2.2 kg). The powdered Mate extract contained <0.5% caffeine and >20.0% of the following six quinic acid esters: 3-caffeoylquinic acid, 4-caffeoylquinic acid, 5-caffeoylquinic acid, 3,5-dicaffeoylquinic acid, 3,4-dicaffeoylquinic acid, and 4,5-dicaffeoylquinic acid. The powdered Mate extract was stored at room temperature along with a desiccant. As previously reported, the optimal dose of Mate administered to SD rats used in *the vivo* experiments was 0.5 g/kg body weight per day. Mate extract was mixed with drinking water to obtain a suspension. For *in vitro* experiments, Mate was dissolved in dimethyl sulfoxide (DMSO) and adjusted to final concentrations of 10, 50, and 100 µg/mL.

### Experimental animals and mate supplementation

Twenty-eight 6-week-old male SD rats were purchased from CLEA Japan, Inc. (Tokyo, Japan). These rats were maintained at 22–24°C under a 12/12 h light–dark cycle. CE-2 (standard diet [340.2 kcal/100 g) (CLEA Japan Inc., Tokyo, Japan)) was provided for an acclimatization period lasting 2 weeks. The SD rats were randomly divided into two groups as follows: 1) Control group (HFD32 [507.6 kcal/100 g) (CLEA Japan Inc., Tokyo, Japan) (Table S1), normal water given by gavage, *n* = 10); and 2) Mate group (HFD32, 0.5 g/kg body weight per day Mate given by gavage, *n* = 8); treatment was administered for 8 weeks. During the administration period, body weight, food intake, and water intake were measured once a week, and blood was collected once a month.

To eliminate the effects of the fasting period, the SD rats were fed for several days after 8 weeks of blood collection to confirm that they had gained weight steadily and returned to their pre-fasting state. The rats dissected after fasting again 12–18 h and sacrificed. SD rats were anesthetized using undiluted isoflurane (FUJIFILM Wako Pure Chemical Industries, Ltd., Osaka, Japan) and soaked in defatted cotton. Dissections were performed by skilled researchers with utmost care and an effort to minimize pain in the laboratory animals. Abdominal circumference was measured, and blood samples were obtained from the abdominal aorta; moreover, plasma and serum samples were collected via centrifugation and cryopreserved at −20°C for future experiments. The liver, kidneys, heart, spleen, brain, EAT, perirenal adipose tissue (PAT), mesenteric adipose tissue (MAT), and subcutaneous adipose tissue (SAT) were removed, and their weights were measured. The dissected organs and tissues were immediately frozen in liquid nitrogen and stored at −80°C for use in western blotting and real-time polymerase chain reaction (PCR). Some of the dissected organs and tissues were preserved in 4% formalin (Nacalai Tesque Inc., Kyoto, Japan) for use in histological analysis.

### Blood metabolic parameter analysis

Fasting blood glucose (FBG) levels were measured using blood samples collected from the tail vein via a self-tested glutest sensor (Sanwa Chemical Research Institute, Aichi, Japan). The levels of serum TG, TC, non-esterified fatty acid (NEFA), aspartate aminotransferase (AST), and alanine aminotransferase (ALT) were measured using a commercially available assay kit (FUJIFILM Wako Pure Chemical Industries, Ltd., Osaka, Japan).

### 3T3-L1 adipocytes culture and treatment

Mouse 3T3-L1 adipocytes were cultured at 37°C under 5% CO_2_ enriched air in 1.0 g/L glucose Dulbecco’s modified eagle medium (DMEM) (Nacalai Tesque Inc., Kyoto, Japan) supplemented with 10% fetal bovine serum (FBS) (Nichirei biosciences Inc., Tokyo, Japan) and 1% penicillin–streptomycin (Nacalai Tesque Inc., Kyoto, Japan). To investigate the effect of Mate extract on intracellular lipid accumulation, confluent 3T3-L1 adipocytes were treated with high glucose DMEM culture medium containing 4.5 g/L glucose (Nacalai Tesque Inc., Kyoto, Japan), 5 µg/mL insulin (Nacalai Tesque Inc., Kyoto, Japan), 500 µM 3-isobutyl-1-methylxanthin (IBMX) (Nacalai Tesque Inc., Kyoto, Japan), and 1 µM dexamethasone (DEX) (Nacalai Tesque Inc., Kyoto, Japan) in the presence or absence of 10, 50, and 100 µg/mL Mate extract solution. After 3 days, the culture medium was replaced with fresh medium supplemented with 5 µg/mL insulin with or without the Mate extract, and the adipocytes were cultured for another 2 days. To further culture the adipocytes, the medium with or without the Mate extract solution was replaced with fresh medium, and the adipocytes were cultured for 2 days. Full differentiation was achieved on day 7. For experiments with the inhibitor, 3T3-L1 adipocytes were treated with methylisobutylxanthine, dexamethasone, and insulin (MDI) culture medium without Mate for 7 days. Following differentiation, 3T3-L1 adipocytes were treated with or without 10 µM dorsomorphin, an AMPK inhibitor (Santa Cruz Biotechnology, Texas, USA), and Mate for 48 h. Subsequently, Oil red O staining was performed, and proteins were extracted (10, 59, 60).

### Cell viability assay

The viability of 3T3-L1 adipocytes was assessed using the methylthiazolyl tetrazolium (MTT) assay. After 7 days of stimulation with MDI medium and Mate, 5 mg/mL MTT solution (Nacalai Tesque Inc., Kyoto, Japan) was added to the cultured medium and incubated at 37°C for 3 h. The culture medium was removed, and reaction products were suspended in DMSO. Cell viability was calculated by measuring the absorbance of each well at 570 nm using a microplate reader (Biotek, Tokyo, Japan) (61).

### Oil red O staining

Differentiated 3T3-L1 adipocytes were washed with phosphate-buffered saline (PBS) and fixed in 10% formalin (Nacalai Tesque Inc., Kyoto, Japan) for 10 min. The fixation solution was removed and discarded, and the 3T3-L1 adipocytes were washed with PBS and incubated with 60% isopropanol (FUJIFILM Wako Pure Chemical Industries, Ltd., Osaka, Japan) for 1 min. The adipocytes were then incubated with Oil red O staining solution (Nacalai Tesque Inc., Kyoto, Japan) for 20 min. The staining solution was removed, and the adipocytes were washed with 60% isopropanol and PBS. Oil red O stained the lipid droplets, which were dissolved in 100% isopropanol (FUJIFILM Wako Pure Chemical Industries, Ltd., Osaka, Japan), and absorbance was measured at 540 nm using a microplate reader (Biotek, Tokyo, Japan) to evaluate intracellular lipid accumulation (1, 10).

### Protein isolation experiments

Proteins were extracted from EAT, PAT, MAT, SAT, and 3T3-L1 adipocytes using a homogenized buffer containing the following: 50 mM Tris-HCl (pH 7.4), 100 mM NaCl, 1% NP-40, 0.25% sodium deoxycholate, 0.1% SDS, 1 mM EDTA, 50 mM NaF, 2 mM Na_3_VO_4_, 30 mM sodium pyrophosphate, and 2 mM PMSF. The extracted proteins were incubated on ice for 30 min and centrifuged at 12,000 rpm for 10 min; the supernatants were stored as extracted samples. Next the extracted proteins were mixed with an equal amount of 2× SDS sample buffer (0.5 M Tris-HCl (pH 6.8), glycerol, SDS, 1% bromophenol blue, and 2-mercaptoethanol) and heated at 100°C for 2.5 min. These samples were used for western blotting analysis (62, 63).

### Primary and secondary antibodies

Primary and secondary antibodies against anti-rabbit AMPK, anti-rabbit phosphor-AMPK, anti-rabbit ACC, anti-rabbit phosphor ACC, anti-rabbit phosphor-hormone sensitive lipase (HSL), anti-rabbit FAS, anti-mouse glucose transporter 4 (GLUT4), anti-rabbit LKB1, anti-rabbit phosphor-LKB1, anti-rabbit CaMKK, anti-rabbit phosphor-CaMKK, anti-rabbit protein kinase A (PKA), anti-rabbit phosphor PKA, anti-rabbit Sirtuin 1 (Sirt1), anti-rabbit PPARγ, anti-rabbit C/EBPα, anti-rabbit C/EBPβ, anti-rabbit insulin receptor substrate 1 (IRS1), anti-rabbit phosphor IRS1 (Ser1101), anti-rabbit phosphor Erk, anti-rabbit uncoupling protein 1 (UCP1), anti-rabbit mammalian target of rapamycin (mTOR), anti-rabbit phosphor-mTOR, anti-rabbit phosphor ribosomal protein S6 kinase (S6K), anti-rabbit glyceraldehyde-3-phosphate dehydrogenase (GAPDH), anti-rabbit IgG, and anti-mouse IgG were purchased from Cell Signaling Technology (Commonwealth of Massachusetts, USA). Anti-rabbit phosphor C/EBPβ was purchased from Abcam (Cambridge, UK); HSL was purchased from Sigma (State of Missouri, U.S.A.); and anti-rabbit IRβ, anti-rabbit phosphor IRβ, and anti-rabbit phosphor IRS1 (Tyr465) were purchased from Santa Cruz Biotechnology (Texas, USA).

### Western blotting

Proteins (10–25 *µ*g/lane) were electrophoresed at 100 V for 1.5–2 h using a 10–12.5% sodium dodecyl sulfate- polyacrylamide gel electrophoresis (SDS–PAGE) gel. The proteins were then transferred onto a polyvinylidene difluoride (PVDF) membrane (Amersham Life Science Inc., Commonwealth of Massachusetts, USA) at 100 mA for 2 h. The membranes were incubated with Blocking One or Blocking One-P solution (Nacalai Tesque Inc., Kyoto, Japan) for 30 min at room temperature and then treated with the primary antibody in Can Get Signal Solution 1 (1:1000 dilution) (TOYOBO CO., LTD., Osaka, Japan). After incubating overnight at 4°C, the membranes were washed with Tris-buffered saline with Tween^®^20 (TBST) containing 1 M Tris-HCl (pH7.5), NaCl, and 20% Tween^®^20. The membranes were then incubated with anti-rabbit or -mouse horseradish peroxidase-conjugated IgG with the secondary antibody in Can Get Signal Solution 2 (1:10000–2000 dilution) (TOYOBO CO. LTD., Osaka, Japan) at room temperature for 1 h. Subsequently, the membranes were washed with TBST, and the protein bands were detected using Chemi-Lumi One Super (Nacalai Tesque Inc., Kyoto, Japan) and Ez-Capture ST (ATTO Corporation, Tokyo, Japan). The levels of protein expression were evaluated. GAPDH was used as an internal control. Protein band intensity analysis was performed using Image J Public Domain Software (National Institutes of Health, State of Maryland, USA) (62, 63).

### RNA extraction and real-time PCR

RNA was extracted from EAT and 3T3-L1 adipocytes using Sepasol (R)-RNA I Super G (Nacalai Tesque Inc., Kyoto, Japan). Absorbance of the extracted samples was measured at 260 and 280 nm using a branch photometer (Hitachi Ltd., Tokyo, Japan) to determine the RNA concentration. We performed reverse transcription to synthesize cDNA using Rever Tra Ace qPCR RT Master Mix and gDNA Remover (TOYOBO CO., LTD., Osaka, Japan). Then, real-time PCR was performed using THUNDERBIRD Next SYBR qPCR Mix (TOYOBO LTD., Osaka, Japan) and was used to determine the relative expression levels of each gene. For real-time PCR, we used specific primers synthesized by Thermo Fisher Scientific (Massachusetts, USA) (Table S2). RNA amplification was performed using a Thermal Cycler Dice (Takara Bio Co. Ltd., Shiga, Japan) based on the following protocol: 95°C for 30 s, 95°C for 5 s, and 60°C for 30 s. The relative expression levels of mRNA were determined using GAPDH as a reference housekeeping gene. The ratio of each transcript was calculated using the 2^−ΔΔCt^ method (63, 64).

### Specific primer sequences

The specific primers of the following genes were used for real-time PCR: *Gapdh*, *Srebp1c*, adipose TG lipase (*Atgl*), adipocyte protein 2 (*ap2*), carnitine palmitoyltransferase 1 (*Cpt1*), *Ucp1*, acyl-CoA oxidase (*Aco*), middle-chain acyl-CoA dehydrogenase (*Mcad*), *Ppar*α, fatty acid-binding protein 4 (*Fabp4*), *Tnf*α, *Il6*, interleukin 1β (*Il1b*), *Adiponectin*, and *Leptin*. All primers were purchased from Thermo Fisher Scientific (Commonwealth of Massachusetts, USA) (Table S2).

### Statistical analysis

All experimental data are expressed in terms of mean ± standard error. Statistical analyses were performed using Student’s *t*-test and one-way ANOVA. Statistical significance was set at *p* < 0.05. Only animals were used in our analysis.

## Results

### Effects of Mate on body weight, body weight gain, food intake, water intake, calorie intake, organ and tissue weights, and abdominal circumference in HFD-fed obese SD rats

**Fig. 1:**
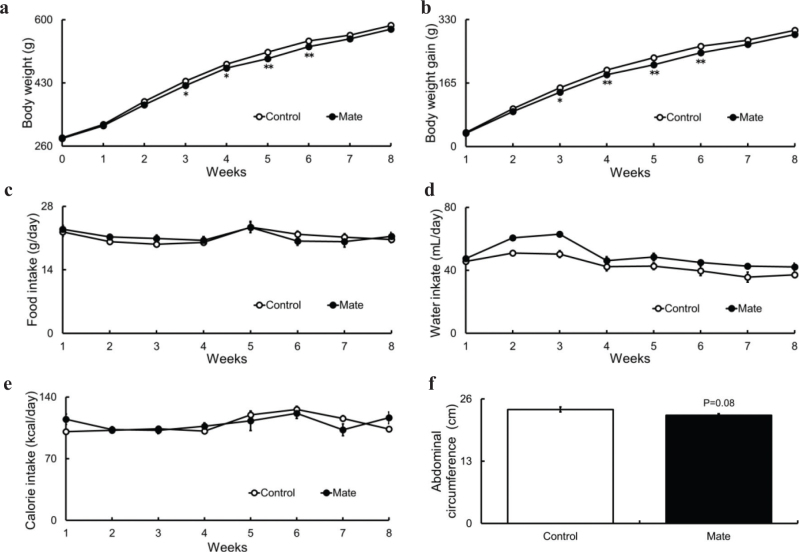
Body weight; body weight gain; abdominal circumference; and food, water, and calorie intake in HFD-fed obese SD rats for 8 weeks under Control or Mate supplementation. Mate (0.5 g/kg body weight/day) reduced body weight, body weight gain, and abdominal circumference in HFD-fed obese SD rats. (a) Body weight, (b) body weight gain, (c) food intake, (d) water intake, (e) calorie intake, and (f) abdominal circumference. White bars represent the Control group, and black bars represent the Mate group. The data are represented as means ± SEM (*n* = 10, 10, and 8, respectively), *P < 0.05, **P < 0.01 versus the Control group.

**Fig. 2:**
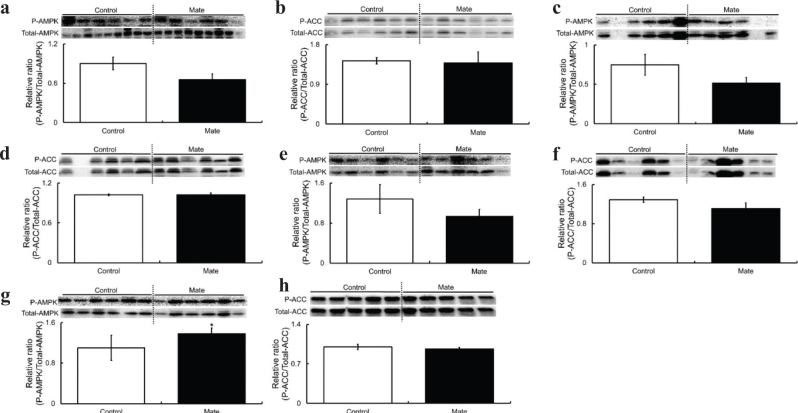
Phosphorylation levels of AMPK and ACC in PAT, MAT, SAT, and EAT of HFD-fed obese SD rats for 8 weeks under Control or Mate supplementation. Mate (0.5 g/kg body weight/day) increased AMPK phosphorylation levels in the EAT of HFD-fed obese SD rats. (a) AMPK of PAT, (b) ACC of PAT, (c) AMPK of MAT, (d) ACC of MAT, (e) AMPK of SAT, (f) ACC of SAT, (g) AMPK of EAT, and (h) ACC of EAT. White bars represent the Control group, and black bars represent the Mate group. The data are represented as means ± SEM (*n* = 10, 10, and 8, respectively), *P < 0.05, **P < 0.01 versus the Control group.

First, we investigated the effects of Mate on body weight, body weight gain, food intake, water intake, calorie intake, organ and tissue weights, and abdominal circumference in HFD-fed obese SD rats. When Mate or water was administered to SD rats for 8 weeks, both body weight and body weight gain tended to be lower at 2 weeks after the start of treatment (*P* = 0.06) and significantly lower after 3 weeks (Fig. 1a, b). No differences were observed in food, water, and calorie intake in the Mate group (Fig. 2c–e). Compared with those of the control group, the weights of the liver and brain in the Mate group were significantly reduced, but the weight of other organs and tissues remained unchanged (Table S3). Furthermore, the abdominal circumference in the Mate group showed a tendency to decrease (Fig. 1f).

### Effects of mate on blood metabolic parameters in HFD-fed obese SD rats

The blood metabolism parameters of HFD-fed obese SD rats were evaluated. Serum TC levels decreased significantly 8 weeks after the start of Mate administration. FBG level was found to be decreased 4 and 8 weeks after administration, although these changes were not significantly different (*P* = 0.05 and 0.06, respectively). The serum levels of TC, TG, NEFA, AST, and ALT were not affected by Mate treatment (Table 1).

**Table 1:** Blood metabolic parameters in HFD-fed obese SD rats for 0, 4, and 8 weeks under Control or Mate supplementation

Blood parameters	Week	Control	Mate
FBG (mg/dL)	0	74.0 ± 4.00	76.3 ± 3.61
	4	93.3 ± 2.70	85.5 ± 2.60
	8	170.2 ± 6.93	141.6 ± 13.34
TC (mg/dL)	0	56.5 ± 3.07	53.9 ± 3.39
	4	112.0 ± 8.08	91.9 ± 8.59
	8	104.2 ± 6.14	83.3 ± 6.03*
TG (mg/dL)	0	60.8 ± 4.80	56.1 ± 5.08
	4	92.1 ± 9.01	101.2 ± 10.69
	8	81.1 ± 12.5	88.6 ± 9.54
NEFA (mEq/L)	0	1.27 ± 0.14	1.23 ± 0.08
	4	0.66 ± 0.04	0.85 ± 0.12
	8	0.45 ± 0.03	0.49 ± 0.05
AST (IU/L)	0	82.1 ± 6.97	72.9 ± 4.04
	4	69.6 ± 5.34	63.3 ± 8.13
	8	42.6 ± 6.80	41.1 ± 3.98
ALT (IU/L)	0	6.12 ± 0.63	4.48 ± 0.69
	4	11.49 ± 1.57	10.08 ± 0.63
	8	5.93 ± 0.60	5.61 ± 0.64

Mate (0.5 g/kg body weight/day) decreased serum TC level in HFD-fed obese SD rats. The table shows the levels of FBG, TC, TG, NEFA, ASL, and ALT. The data are represented as means ± SEM (*n* = 10, 10, and 8, respectively),

* *P* < 0.05 versus the Control group.

### Effects of mate on the phosphorylation levels of AMPK and ACC in PAT, MAT, SAT, and EAT of HFD-fed obese SD rats

To elucidate the mechanism underlying the suppression of body weight gain and reduction in serum TC levels of HFD-fed obese SD rats treated with Mate, we evaluated four different types of adipose tissues (PAT, MAT, SAT, and EAT) that play a key role in lipid metabolism. We investigated the phosphorylation levels of AMPK and ACC, which play a key role in the lipid metabolism pathway.

Mate treatment did not affect the phosphorylation levels of AMPK and ACC in PAT (Fig. 2a, b), MAT (Fig. 2c, d), and SAT (Fig. 2e, f). However, the AMPK phosphorylation levels of EAT were significantly increased although ACC phosphorylation levels remained unchanged (Fig. 2g, h). These results indicated that EAT activates AMPK, and thus, we selected EAT for use in further studies aimed at investigating the mechanisms underlying the prevention of body weight gain and the reduction in serum TC levels by Mate, as well as the expression levels of other factors involved in lipid metabolism pathways. Another reason for selecting EAT was that it had been used in numerous studies as an indicator of visceral adipose tissue.

### Effects of mate on the phosphorylation and expression levels of AMPK-dependent pathway- and insulin signaling pathway-related proteins in the EAT of HFD-fed obese SD rats

**Fig. 3:**
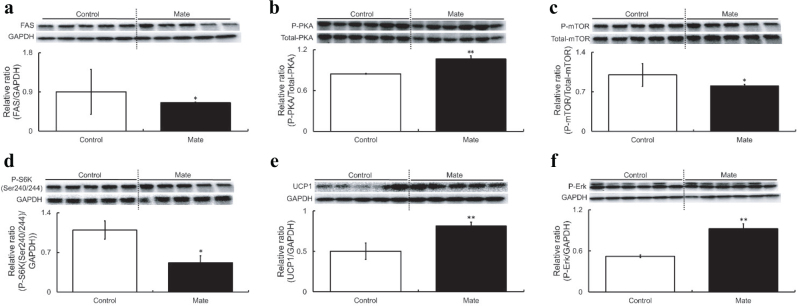
Phosphorylation and expression levels of FAS, PKA, mTOR, S6K, UCP1, and Erk in the EAT of HFD-fed obese SD rats for 8 weeks under Control or Mate supplementation. Mate (0.5 g/kg body weight/day) increased PKA phosphorylation and UCP1 expression levels and decreased mTOR and S6K phosphorylation levels in the EAT of HFD-fed obese SD rats. (a) FAS, (b) PKA, (c) mTOR, (d) S6K, (e) UCP1, and (f) Erk. White bars represent the Control group, and black bars represent the Mate group. The data are represented as means ± SEM (*n* = 10, 10, and 8, respectively), *P < 0.05, **P < 0.01 versus the Control group.

As AMPK activation was observed in the EAT of SD rats treated with Mate, the phosphorylation and expression levels of proteins associated with the AMPK-dependent and insulin signaling pathways in EAT were investigated. First, we assessed the phosphorylation and expression levels of HSL, FAS, and GLUT4, which are downstream regulators of AMPK. Although no change was observed in the levels of HSL phosphorylation or GLUT4 expression (data not shown), but FAS expression in the Mate group was found to be significantly reduced (Fig. 3a). Next, we investigated the activation of CaMKK, LKB1, Sirt1, and PKA, which are upstream regulators of AMPK. The levels of CaMKK and LKB1 phosphorylation and Sirt1 expression in the Mate group were not altered (data not shown), but PKA was significantly activated (Fig. 3b). Reportedly, mTOR and S6K are other downstream factors of AMPK. Inactivation of mTOR and S6K inhibits the pathways associated with adipogenesis (64). Mate treatment reduced the phosphorylation levels of mTOR and S6K (Fig. 3c, d). Furthermore, the expression level of UCP1, a browning-specific transcription factor, was significantly increased in the Mate group (Fig. 3e). We examined the phosphorylation and expression levels of IRβ, IRS1 (Ser1101), IRS1 (Tyr465), Erk, PPARγ, C/EBPα, and C/EBPβ, which are proteins and transcription factors related to the insulin signaling pathway, and found that Erk phosphorylation was significantly increased by Mate (Fig. 3f); however, there were no significant differences between the other protein in the control and Mate groups (data not shown).

### Effects of Mate on the expression levels of lipid metabolism-related genes in the EAT of HFD-fed obese SD rats

We investigated the effect of Mate on the expression levels of genes related to lipid metabolism in the EAT of HFD-fed obese SD rats. Real-time PCR was used to examine the relative expression levels of *Serbp1c*, *Atgl*, *ap2*, *Cpt1*, *Ppara*, *Fabp4*, *Aco*, *Mcad*, *Tnfa*, *Il6*, *Il1b*, *Adiponectin*, *Leptin*, and *Ucp1*. Mate treatment did not affect the mRNA expression levels of *Atgl*, *Cpt1*, *Ppara*, *Aco*, *Mcad*, *Tnfa*, and *Il1b* (data not shown). However, the mRNA expression levels of *Srebp1c* and *ap2*, which encode downstream components of AMPK, as well as *Adiponectin* and *Leptin*, which are involved in lipogenesis, were significantly reduced in the Mate group. Moreover, the expression levels of *Il6*, an inflammatory cytokine, and *Fabp4*, an index of body fat mass, were significantly reduced by Mate. In addition, the *UCP1* mRNA level in the Mate group was significantly increased, a result which was consistent with that of the protein analysis (Table 2).

**Table 2:** mRNA expression levels of *Srebp1c*, *ap2*, *Fabp4*, *Il6*, *Adiponectin*, *Leptin*, and *Ucp1* in the EAT of HDF-fed obese SD rats for 8 weeks under Control or Mate supplementation

Genes	Control (%)	Mate (%)
*Srebp1c*	100.0 ± 0.49	26.2 ± 0.67*
*ap2*	100.0 ± 0.27	25.6 ± 0.35**
*Fabp4*	100.0 ± 0.31	28.6 ± 0.03*
*Il6*	100.0 ± 0.28	9.68 ± 0.98*
*Adiponectin*	100.0 ± 0.46	12.8 ± 0.77*
*Leptin*	100.0 ± 0.54	30.5 ± 0.40*
*Ucp1*	100.0 ± 0.70	1218.9 ± 0.60**

Mate (0.5 g/kg body weight/day) increased the gene expression level of *Ucp1* and decreased the gene expression levels of *Srebp1c*, *ap2*, *Fabp4*, *Il6*, *Adiponectin*, and *Leptin* in the EAT of HFD-fed obese SD rats. The table shows the genes expression levels of *Srebp1c*, *ap2*, *Cpt1*, *Fabp4*, *Il6*, *Adiponectin*, *Leptin*, and *Ucp1*. The data are represented as means ± SEM (*n* = 10, 10, and 8, respectively),

* *P* < 0.05,

** *P* < 0.01 versus the Control group.

### Effects of Mate on cell viability and intracellular lipid accumulation in 3T3-L1 adipocytes

**Fig. 4:**
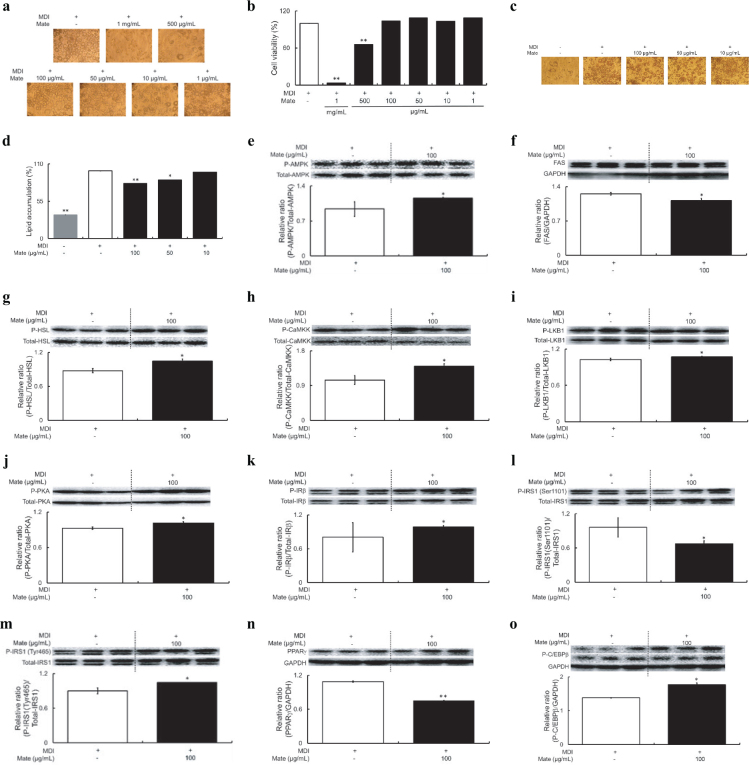
Cell viability, intracellular lipid accumulation effects, and phosphorylation and expression of AMPK, HSL, FAS, CaMKK, LKB1, PKA, IRβ, IRS1 (Ser1101), IRS1 (Tyr465), PPARγ, and C/EBPβ in MDI-treated 3T3-L1 adipocytes for 7 days under Control or Mate supplementation. Mate (10, 50, or 100 µg/mL) did not affect cell viability, reduced intracellular lipid accumulation, increased the phosphorylation levels of AMPK, HSL, CaMKK, LKB1, PKA, and C/EBPβ, and decreased the expression levels of FAS and PPARγ in MDI-treated 3T3-L1 adipocytes. (a) Cell viability 7 days after induction with MDI medium (100× magnification), (b) quantitative analysis of cell viability, (c) Oil red O staining 7 days after induction with MDI medium (100× magnification), (d) quantitative analysis of Oil red O staining, (e) AMPK, (f) HSL, (g) FAS, (h) CaMKK, (i) LKB1, (j) PKA, (k) IRβ, (l) IRS1 (Ser1101), (m) IRS1 (Tyr465), (n) PPARγ, and (o) C/EBPβ. Gray bars represent the MDI (non-treated) group, white bars represent the Control (treatment with MDI alone) group, and black bars represent the Mate (treatment with both MDI and Mate) group. The data are represented as means ± SEM (*n* = 3), *P < 0.05, **P < 0.01 versus the Control group.

First, we examined the effect of Mate on intracellular lipid accumulation in 3T3-L1 adipocytes. To determine cell viability, 3T3-L1 adipocytes were treated with the following concentrations of Mate (1, 10, 50, 100, and 500 µg/mL, and 1 mg/mL (final concentration)). Following stimulation with MDI medium for 7 days, cell viability was measured using an MTT assay. The viability of 3T3-L1 adipocytes was significantly reduced 500 µg/mL and 1 mg/mL of Mate (Fig. 4a, b). Therefore, we selected the concentrations of 10, 50, and 100 µg/mL for uses in subsequent cell experiments, in order to avoid the cytotoxic range of Mate. Intracellular lipid accumulation in 3T3-L1 adipocytes treated with Mate was evaluated via the same procedure uses to conduct the MTT assay, followed by the staining of lipid droplets with Oil red O and measuring absorbance at 540 nm. Although the proportion of lipid droplets stained with Oil red O was in the adipocytes treated with MDI medium alone was significantly increased, it was reduced by Mate treatment in a concentration-dependent manner. In particular, Mate concentrations of 50 and 100 µg/mL markedly suppressed intracellular lipid accumulation in the MDI medium (Fig. 4c, d).

### Effects of Mate on the phosphorylation and expression of AMPK-dependent pathway- and insulin signaling pathway-related proteins in MDI-treated 3T3-L1 adipocytes

To clarify the mechanism underlying the suppression of intracellular lipid accumulation in MDI-induced and Mate-treated 3T3-L1 adipocytes, the phosphorylation and expression levels of AMPK-dependent pathway- and insulin signaling pathway-related proteins were investigated. For protein analysis, we used Mate at a concentration of 100 µg/mL, which had no effect on cell viability but exerted a most significant effect on intracellular fat accumulation suppression, as shown by Oil red O staining. Phosphorylation of AMPK, an important factor in lipid metabolism, was significantly increased in the Mate group (Fig. 4e). The expression levels of ACC, HSL, and FAS, which are downstream regulators of AMPK, were measured. Mate did not change the level of ACC phosphorylation (data not shown) but significantly increased HSL phosphorylation and decreased FAS expression (Fig. 4f, g). In addition, the expression levels of CaMKK, LKB1, Sirt1, and PKA, which are upstream regulators of AMPK, were investigated. It was found that although the expression level of Sirt1 in the Mate group had not changed (data not shown), the phosphorylation levels of CaMKK, LKB1, and PKA in this group had significantly increased (Fig. 4h–j). In addition, an investigation found no differences between the phosphorylation levels of mTOR and S6K, which are other downstream factors of AMPK (data not shown). We also examined the phosphorylation and expression levels of IRβ, IRS1 (Ser1101), IRS1 (Tyr465), Erk, PPARγ, C/EBPα, and C/EBPβ, which are proteins and transcription associated with the insulin signaling pathway. The expression levels of Erk and C/EBPα were not altered (data not shown); however, the phosphorylation and expression levels of IRβ, IRS1 (Tyr465), and C/EBPβ were increased by Mate treatment, while those of IRS1 (Ser1101) and PPARγ were reduced by it (Fig. 4k–o).

### Effects of mate on the expression levels of lipid metabolism-related genes in MDI-treated 3T3-L1 adipocytes

The effect of Mate on the expression levels of genes related to lipid metabolism was investigated using 3T3-L1 adipocytes treated with MDI. We used real-time PCR analysis to determine the expression levels of *Srebp1c*, *ap2*, *Ppara*, *Tnfa*, and *Leptin*. Although Mate treatment did not affect the mRNA levels of *ap2*, *Ppara*, *Tnfa*, and *Leptin*, the expression levels of *Srebp1c*, a downstream factor of AMPK, were found to be significantly decreased (Table 3).

**Table 3:** mRNA expression levels of *Srebp1c*, *ap2*, *Ppara*, *Tnfa*, and *Leptin* in MDI-treated 3T3-L1 adipocytes for 7 days under Control or Mate supplementation

Genes	Control (%)	Mate 100 µg/mL (%)
*Srebp1c*	100.0 ± 0.01	21.1 ± 0.45*
*ap2*	100.0 ± 0.01	102.1 ± 0.17
*Ppara*	100.0 ± 0.12	72.4 ± 0.19
*Tnfa*	100.0 ± 0.63	154.2 ± 0.18
*Leptin*	100.0 ± 0.71	82.1 ± 0.31

Mate (100 µg/mL) decreased the gene expression levels of *Srebp1c* in MDI-treated 3T3-L1 adipocytes. The table shows the genes expression levels of *Srebp1c*, *ap2*, *Ppara*, *Tnfa*, and *Leptin*. The data are represented as means ± SEM (*n* = 3),

* *P* < 0.05 versus the Control group.

### Effects of mate on the AMPK-dependent pathway in MDI-treated 3T3-L1 adipocytes

**Fig. 5:**
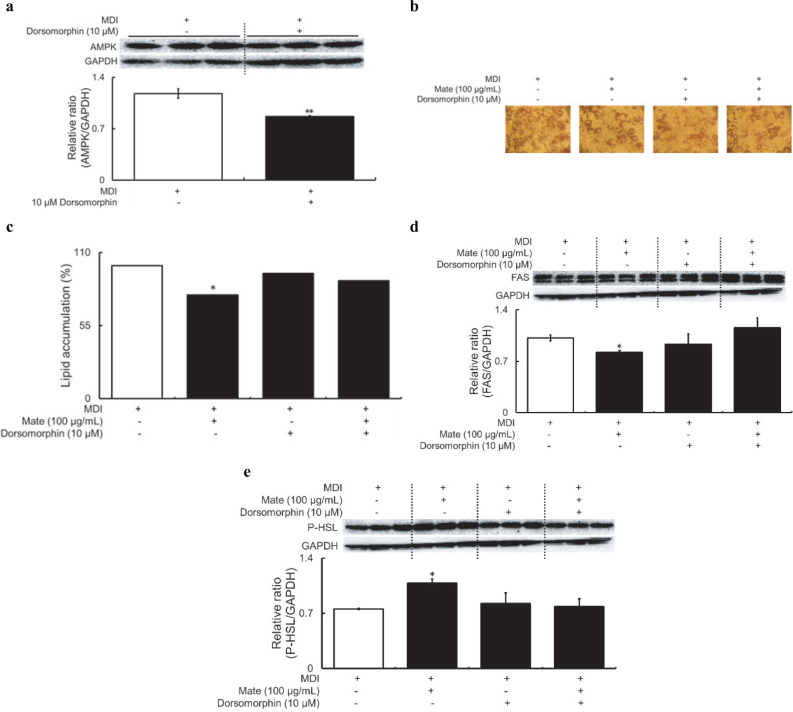
Intracellular lipid accumulation and expression and phosphorylation of FAS and HSL in 3T3-L1 adipocytes for 48 h under dorsomorphin, Control, or Mate supplementation after 7 days MDI treatment. Dorsomorphin (10 µM) inhibited the effect of Mate (100 µg/mL) treatment on intracellular lipid accumulation suppression, and FAS expression and HSL phosphorylation levels were increased and decreased, respectively. (a) AMPK, (b) Oil red O staining 7 days and 48 h after induction with MDI medium and dorsomorphin (100× magnification), (c) quantitative analysis of Oil red O staining, (d) FAS, and (e) HSL. White bars represent the Control (treatment with MDI alone) group, and black bars represent the treatment with MDI and presence or absence of dorsomorphin or Mate group. The data are represented as means ± SEM (*n* = 3), *P < 0.05, **P < 0.01 versus the Control group.

This study indicated that the AMPK-dependent pathway was activated in Mate-treated 3T3-L1 adipocytes in MDI medium. Therefore, we used dorsomorphin, which specifically recognizes and inhibits AMPK, to evaluate whether the action of Mate on the suppression of intracellular lipid accumulation depends on the AMPK-dependent pathway. When 3T3-L1 adipocytes were cultured in MDI medium for 7 days and stimulated with 10 µM dorsomorphin for 48 h, the expression level of AMPK was significantly reduced, indicating successful inhibition of AMPK in the 3T3-L1 adipocytes model (Fig. 5a). The effect of dorsomorphin bases stimulation on intracellular lipid accumulation was investigated using Oil red O staining. After 7 days of differentiation in MDI medium, treatment with 100 µg/mL Mate alone for 48 h reduced intracellular lipid accumulation, but stimulation with dorsomorphin alone did not reduce intracellular lipid accumulation. Furthermore, simultaneous stimulation of 3T3-L1 adipocytes with dorsomorphin and Mate did not alter intracellular lipid accumulation, and the effect of Mate treatment alone ceased (Fig. 5b, c). In addition, FAS expression and HSL phosphorylation in dorsomorphin-stimulated AMPK inhibition in the Mate-treated 3T3-L1 adipocytes were, respectively, increased and decreased compared to that in the cells treated with Mate alone (Fig. 5d–g). These results indicated that the stimulation of 3T3-L1 adipocytes using dorsomorphin eliminated the effect of everted by Mate on FAS and HSL expression levels. Thus, the reduction in intracellular lipid accumulation in the 3T3-L1 adipocytes caused by Mate treatment in 3T3-L1 adipocytes appears to be regulated via the AMPK-dependent pathway.

## Discussion

In this study, we observed that Mate improves lipid metabolism in HFD-fed obese SD rats and suppressed intracellular lipid accumulation in MDI-treated 3T3-L1 adipocytes.

Adipose tissue is a key organ that regulates lipid metabolism pathways, associated with lipid distribution and energy metabolism (66). Adipocytes play an important role in the regulation of lipid metabolism (59). The differentiation of preadipocytes into adipocytes is regulated by a complex process involving the activation of genes, transcription factors, and enzymes involved in adipogenesis. Currently, studies are focusing on the search for safe and novel natural resources that can be used to treat obesity (67–69). Thus, we investigated the effects of Mate on the lipid metabolism-related proteins and genes in the EAT of HFD-fed obese SD rats and 3T3-L1 adipocytes.

First, we investigated the effect of Mate on lipid metabolism in the EAT of HFD-fed obese SD rats. Following treatment with Mate for 8 weeks, body weight gain became significantly lower. Next in order to clarify the mechanism underlying its effect on body weight gain, we analyzed lipid metabolism-related proteins and genes.

AMPK, which acts as a metabolic energy sensor, regulates lipid metabolism and homeostasis (64, 70, 71). The activation of AMPK in adipose tissue by various extracellular stimuli provides a promising molecular target for treating metabolic disorders such as obesity (4). Treatment with Mate significantly activated AMPK, which, in turn, increased and decreased the level of phosphorylation as well as expression of various proteins and genes associated with lipid metabolism via the AMPK-dependent pathway. *Srebp1c* is a lipogenic transcription factor involved in FAS expression and thereby to stimulate fatty acid synthesis (72, 73). Several studies have demonstrated that the phosphorylation of AMPK reduces FAS expression (74–76). PKA is an upstream regulator of LKB1 and *ap2* and LKB1 (58, 77), which stimulates adipogenesis (78). Our results indicated that Mate had increased the phosphorylation of AMPK and PKA and decreased the expression of *Srebp1c*, FAS, and *ap2*, which are adipogenesis-related factors.

*Fabp4*, which is expressed in adipocytes and macrophages, exhibits deep involvement in inflammation and intracellular lipid metabolism. *Adiponectin* and *Leptin* are also adipogenesis-related genes (79). Mate suppressed the mRNA levels of *Fabp4*, *Adiponectin*, and *Leptin*, indicating that Mate inhibits adipogenesis in the EAT of HFD-fed obese SD rats. Mate treatment reduced *Il6* expression in WAT during the development of obesity and insulin resistance (80). An increase in UCP1 expression increases the conversion of free fatty acids during heat dissipation, thereby reducing the amount of TG in the body. This mechanism offers a target for treatment aimed at reducing obesity (79). In our study, Mate increased both protein and gene expression levels of UCP1, indicating that Mate promotes thermogenesis in the EAT of HFD-fed obese SD rats.

These findings revealed that Mate may improve lipid metabolism by inhibiting lipogenesis via the PKA-AMPK-*Srebp1c*-FAS and *ap2* pathways in the EAT of HFD-fed obese SD rats. In addition, it was shown that Mate may also inhibit lipogenesis and promote thermogenesis by suppressing the expression levels of the inflammatory cytokine, *Il6*, and the adipogenesis-related factors, *Fabp4*, *Adiponectin*, and *Leptin*, and enhancing the expression of UCP1.

The proteins, mTOR, S6K, PPARγ, and C/EBPα, are considered to be essential for the treatment of obesity, and these are negatively regulated by AMPK (77, 81, 82, 83). In addition, the AMPK-mTOR-S6K cascade inhibits the expression levels of downstream FAS and *ap2* (78, 84). These finding indicated that Mate reduces the phosphorylation of mTOR and S6K in the EAT of HFD-fed obese SD rats. Furthermore, although Mate treatment did not alter the expression levels of PPARγ and C/EBPα, which are downstream components of the AMPK-mTOR-S6K pathway, it significantly reduced the expression levels of FAS and *ap2*, which are downstream factors involved in adipogenesis. These results suggest that Mate may also regulate adipogenesis through the PKA-AMPK-mTOR-S6K-FAS and *ap2* pathways.

The insulin signaling pathway is also involved in lipid metabolism (22). Thus, we investigated the activation of IRβ, IRS1 (Ser1101), and IRS1 (Tyr465). The insulin signaling pathway contributes to the expression of C/EBPα and PPARγ (25). Therefore, the insulin signaling pathway is also considered as a potential target in obesity treatment. Our results indicated that the phosphorylation levels of IRβ, IRS1 (Ser1101), and IRS1 (Tyr465) in EAT were not affected by Mate treatment. These results showed that lipid metabolism mediated by the insulin signaling pathway was not involved in the improvement in lipid metabolism seen in Mate-administered HFD-fed obese SD rats.

The dosage of Mate did not affect the levels of the liver function indicators and serum AST and ALT. In addition, no abnormalities were found in the defecation activity of SD rats during the experimental period. Furthermore, we did not make any notable finding in the digestive organs of the dissected SD rats. Therefore, it was concluded that the dosage of Mate uses in this study had not exerted any toxicity or side effects.

Next, we investigated the effect of Mate on intracellular lipid accumulation in 3T3-L1 adipocytes. Researchers frequently use 3T3-L1 adipocytes in HFD animal models that are utilized to study obesity in vivo (22, 65, 85–87). Treatment with Mate for 7 days significantly reduced intracellular lipid accumulation in 3T3-L1 adipocytes in a concentration-dependent manner, and therefore, we subsequently analyzed lipid metabolism-related proteins and genes. Treatment 3T3-L1 adipocytes with Mate significantly phosphorylated AMPK, an important enzyme in the lipid metabolism pathway.

Activation of the lipase, HSL, is regulated by cAMP- dependent PKA and protein phosphatase (88). LKB1, an upstream kinase of AMPK, plays an important role in both glucose and lipid metabolism (4, 58). Moreover, CaMKK, which is located upstream of AMPK, responds to an increase in intracellular Ca^2+^ levels (78). The Mate treatment increased the expression levels of these upstream factors of AMPK in 3T3-L1 adipocytes. Thus, it may be inferred that Mate suppresses intracellular lipid accumulation in 3T3-L1 adipocytes by inhibiting adipogenesis and promoting lipolysis via the PKA-LKB1-AMPK-*Srebp1c*-HSL and FAS pathways.

Other lipid metabolism-related factors, such as PPARγ and C/EBPβ (81), were also evaluated. The expression of PPARγ, a lipogenesis-related transcription factor, was decreased, while the phosphorylation of C/EBPβ, a lipolysis-related transcription factor, was increased in 3T3-L1 adipocytes treated with Mate. Reportedly, the activation of PKA phosphorylates the downstream factor, C/EBPβ, thereby suppressing the expression levels of PPARγ and *Srebp1c* located downstream of C/EBPβ, inhibiting FAS expression and reducing adipogenesis (89). Our results suggest that Mate may suppress adipogenesis by activating the PKA-C/EBPβ-PPARγ and *Srebp1c*-FAS pathways in 3T3-L1 adipocytes.

In addition, we examined whether the effects exerted by Mate on the suppression of intracellular lipid accumulation depends on the insulin signaling pathway. In Mate-treated 3T3-L1 adipocytes, the expression of IRβ and IRS1 (Tyr465) was significantly enhanced, while that of IRS1 (Ser1101) was reduced, indicating that Mate may suppress intracellular lipid accumulation in 3T3-L1 adipocytes by activating not only the AMPK-dependent pathway but also the insulin signaling pathway.

Further validation of these results showed that the reduction in intracellular lipid accumulation and fluctuation in the expression levels of lipid metabolism-related proteins, including HSL and FAS, in 3T3-L1 adipocytes were abolished by supplementation with dorsomorphin, which is a specific AMPK inhibitor. These results suggested that the inhibitory effect of intracellular lipid accumulation in 3T3-L1 adipocytes associated with Mate treatment was mediated by AMPK activation.

Evaluations using the HFD-fed obese SD rats showed that Mate may improve lipid metabolism. Thus, Mate treatment improved lipid metabolism in HFD-fed obese SD rats but suppressed intracellular lipid accumulation in 3T3-L1 adipocytes via AMPK-dependent and insulin signaling pathways.

## Conclusions

Mate improved lipid metabolism in HFD-fed obese SD rats and suppressed intracellular lipid accumulation in 3T3-L1 adipocytes. Mate treatment improved lipid metabolism in the EAT of HFD-fed obese SD rats by inhibiting adipogenesis via the AMPK-dependent, PKA-AMPK-*Srebp1c*-FAS-*ap2* and PKA-AMPK-mTOR-S6K-FAS-*ap2* pathways and promoting thermogenesis by increasing the expression of UCP1. Furthermore, the decrease in adipogenesis was reflected by a decrease in expression levels of *Adiponectin*, *Leptin*, and *Fabp4* in the EAT of HFD-fed obese SD rats treated with Mate (Fig. S1). The improvement in lipid metabolism caused by Mate in turn inhibited the gain in body weight and reduction in serum TC levels seen in HFD-fed obese SD rats. The suppression of intracellular lipid accumulation by Mate may be attributed to the inhibition of adipogenesis and the promotion of lipolysis via the activation of the AMPK-dependent, PKA-LKB1-AMPK-*Srebp1c*-HSL-FAS and PKA-C/EBPβ-PPARγ-*Srebp1c*-FAS pathways (Fig. S2). In addition, Mate was shown to suppress intracellular lipid accumulation in 3T3-L1 adipocytes by reducing the expression levels of the downstream factor, PPARγ, and increasing the phosphorylation level of C/EBPβ via the activation of the insulin signaling pathway. Mate may not only improve metabolic disorders but also help suppress the onset of lifestyle-related diseases, such as hypertension, hyperlipidemia, and diabetes, caused by excessive body weight gain. Being a natural resource, Mate shows potential for use as a new and safe therapeutic agent for the treatment of metabolic diseases, although further clinical studies may be needed prior to its validation as a clinical application.

## Conflict of interest and funding

The authors are not applicable potential conflict of interests and funding.
