# Supplementary material for: Ilex paraguariensis A.St.-Hil. improves lipid metabolism in high-fat diet-fed obese rats and suppresses intracellular lipid accumulation in 3T3-L1 adipocytes via the AMPK-dependent and insulin signaling pathways
Source: Food Nutr Res. 2024 Jan 22;68:10.29219/fnr.v68.10307. doi: 10.29219/fnr.v68.10307 (PMC10845893; doi:10.29219/fnr.v68.10307)
Supplement: Supplementary file 1 [file FNR-68-10307-s001.docx]

**Supplementary materials**

**Table S1 Ratio of each component of the HFD 32**

| Components | % |
| --- | --- |
| Milk casein | 24.500 |
| Albumen powder | 5.000 |
| L-Cystin | 0.430 |
| Beef tallow powder  (Contains 80% beef tallow) | 15.880 |
| Safflower oil (High oleic acid type) | 20.000 |
| Crystalline cellulose | 5.500 |
| Maltodextrin | 8.250 |
| Lactose | 6.928 |
| Sucrose | 6.750 |
| AIN93 vitamin mix | 1.400 |
| AIN93 mineral mix | 5.000 |
| Choline bitartrate | 0.360 |
| 3- Butyl hydroxynon | 0.002 |
| Total | 100.000 |

**Table S2 Specific primer sequences of lipid metabolism-related genes**

| Genes | Forward | Reverse |
| --- | --- | --- |
| *Gapdh* | AGAACATCATCCCTGCATCCA | CCGTTCAGCTCTGGGATGAC |
| *Srebp1c* | GGAGCCATGGATTGCACATT | CCTGTCTCACCCCCAGCATA |
| *Atgl* | GGTGCCAACATTATTGAGGTG | AAACACGAGTCAGGGAGATGC |
| *ap2* | CAAAATGTGTGATGCCTTTGTC | CTCTTCCTTTGGCTCATGCC |
| *Cpt1* | CGGTTCAAGAATGGCATCATC | ATCACACCCACCACCACGATA |
| *Ucp1* | TACAGAGTTATAGCCACCACCACA | TGGAACGTCATCATCATGTTTGTG |
| *Aco* | CCCAAGACCCAAGAGTTCATTC | CACGGATAGGGACAACAAAGG |
| *Mcad* | TGTGCCTACTGCGTGACAGA | TTCATCACCCTTCTTCTCTGCTT |
| *Ppara* | TGGAGTCCACGCATGTGAAG | TGTTCCGGTTCTTTTTCTGAATCT |
| *Fabp4* | CAAGCCCAACATGATCATGAGC | CACGCCCAGTTTGAAGGAAATC |
| *Tnfa* | AAATGGGCTCCCTCTCATCAGTTC | TCTGCTTGGTGGTTTGCTACGAC |
| *I16* | CAGTGTCATGGTTCCTTTGC | CACCGAGGAACTACCTGAT |
| *I11b* | CACCTCTCAAGCAGAGCACAG | GGGTTCCATGGTGAAGTCAAC |
| *Adiponectin* | CAGAATTCATTATGACGGCAGCAC | AGACTTGGTCTCCCACCTCCA |
| *Leptin* | GACATTTCACACAGGCAGTCG | GCAAGCTGGTGAGGATCTGT |

**Table S3 Organ and tissue weights in HFD-fed obese SD rats for 8 weeks under Control or Mate supplementation.**

| Organ and tissue | Control  (mg/g (body weight)) | Mate  (mg/g (body weight)) |
| --- | --- | --- |
| Liver | 27.7±0.95 | 23.3±0.70** |
| Kidneys | 4.83±0.11 | 4.66±0.07 |
| Spleen | 1.55±0.02 | 1.56±0.10 |
| Heart | 2.16±0.03 | 2.16±0.07 |
| EAT | 24.6±2.63 | 24.8±2.76 |
| PAT | 32.2±3.10 | 40.9±1.64 |
| Brain | 2.54±0.05 | 2.67±0.02* |

Mate (0.5 g/kg body weight/day) decreased the weight of the liver and brain in HFD-fed obese SD rats. The table shows the weights of the liver, kidneys, spleen, heart, EAT, PAT, and brain. The data are represented as means ± S.E.M. (n = 10, 10, 8 respectively), **P* < 0.05, ***P* < 0.01 vs. the Control group.

**Fig. S1 Pathway associated with the improvement of lipid metabolism in the EAT of HFD-diet fed obese SD rats for 8 weeks under Control or Mate (0.5 g / kg body weight / day) supplementation.**

**
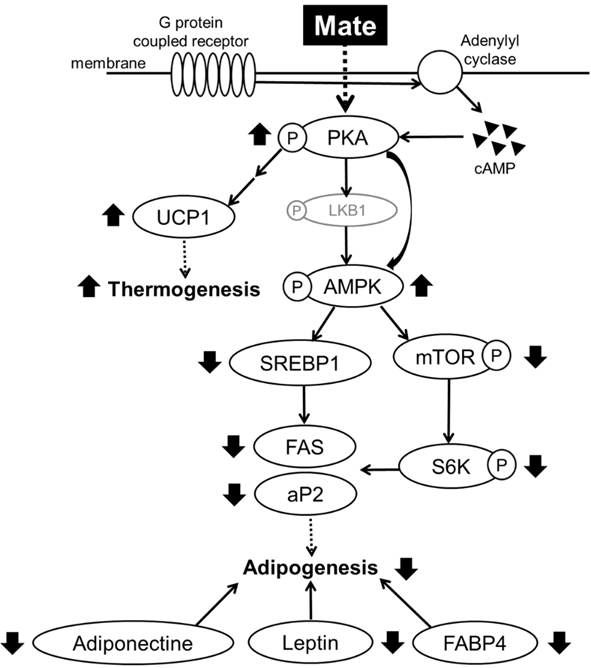
**

**Fig. S2 Pathway associated with the suppression of intracellular lipid accumulation in MDI-induced 3T3-L1 adipocytes for 7 d under Control or Mate (10, 50, 100 µg/mL) supplementation.**

**
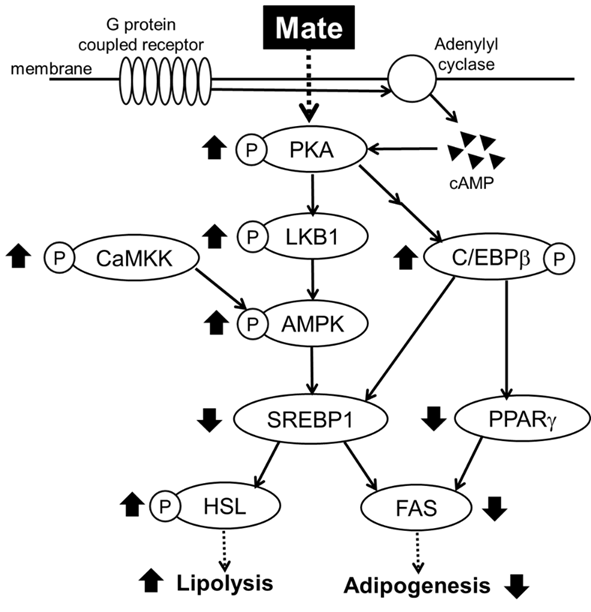
**
